# CD47 is a regulator in doxorubicin‐induced cardiac aging in male mice

**DOI:** 10.14814/phy2.71111

**Published:** 2026-09-25

**Authors:** Aiwei Yan, Rui Mai, Ting Cao, Changen Duan, Sheng Liu, Xianwei Wang

**Affiliations:** ^1^ Department of Cardiovascular Surgery, the First Affiliated Hospital Xinxiang Medical University Weihui China; ^2^ Henan Key Laboratory of Medical Tissue Regeneration Xinxiang Medical University Xinxiang China

**Keywords:** cardiac aging, cardiomyocyte senescence, CD47, doxorubicin

## Abstract

Cardiac aging is a natural process that occurs with age, leading to structural and functional abnormalities of the heart. In addition to chronological age, anticancer therapies can accelerate this process. Doxorubicin, an anthracycline class antitumor drug, is known to have significant cardiac toxicity including cardiac aging, but the underlying mechanisms remain incompletely understood. In this study, we identified that CD47 was significantly increased in aged hearts through bioinformatic analyses. We next established a doxorubicin‐induced cardiac aging model in mice. Doxorubicin increased p53, p16, and p21 expression in mouse hearts, accompanied by marked CD47 upregulation. Similar effects were observed in AC16 cardiomyocytes, in which doxorubicin induced CD47 expression and a senescence‐like phenotype, including increased p16 and p21 levels and SA‐β‐gal positivity. Blocking CD47 with a specific antibody attenuated doxorubicin‐induced cardiac aging and cardiomyocyte senescence in vivo and in vitro, and improved doxorubicin‐induced cardiac dysfunction. These results identify CD47 as a functional mediator of doxorubicin‐induced cardiac aging and support CD47 blockade as a potential strategy to mitigate anthracycline‐associated cardiac dysfunction.

## 1 INTRODUCTION

Heart aging is characterized by several pathological features, including reduced contractility and metabolic efficiency, cardiac fibrosis, and cardiac hypertrophy (Lazzeroni et al., 2022; Wessells & Bodmer, 2007; Yan et al., 2021). These pathological changes contribute to the progressive decline in cardiac function associated with aging, thereby increasing the risk of heart failure and other cardiovascular diseases (Dai et al., 2014; North & Sinclair, 2012). Beyond chronological aging, cancer therapies can also accelerate cardiac aging (Florido et al., 2022). Doxorubicin, a widely used anthracycline chemotherapeutic agent, induces cardiotoxicity, cardiac aging, and cardiomyocyte senescence (Minotti et al., 2004). Mechanistically, doxorubicin can intercalate into DNA, interfere with topoisomerase‐II‐dependent DNA repair, promote DNA damage, and disrupt chromatin organization through nucleosome destabilization and histone eviction (Linders et al., 2024; Pang et al., 2013). It also induces mitochondrial dysfunction, oxidative stress, impaired autophagy and proteostasis, inflammatory activation, and senescence‐associated signaling, thereby contributing to myocardial fibrosis, hypertrophy, metabolic dysfunction, and contractile impairment (Ravindran & Gustafsson, 2025). However, how these upstream injuries evolve into persistent cardiac aging, and whether immune‐checkpoint‐like membrane molecules are involved, remains incompletely defined. Effective strategies to mitigate doxorubicin‐induced cardiac aging are still limited.

CD47 is a transmembrane glycoprotein that is highly expressed in the heart and has emerged as an important regulator of cardiovascular homeostasis and stress response (Gao et al., 2024; Hu et al., 2019; Tan et al., 2024). It interacts with several ligands, including signal regulatory protein alpha (SIRPα), thrombospondin‐1 (TSP‐1), and integrins (Van Duijn et al., 2022). The CD47‐SIRPα axis transmits a “don't eat me” signal by activating inhibitory signaling in macrophages and other myeloid cells, thereby suppressing phagocytosis and efferocytosis (Manara et al., 2024). TSP‐1‐CD47 signaling regulates nitric oxide/cGMP signaling, oxidative stress, vascular tone, angiogenesis, and cell survival, whereas CD47‐integrin interactions influence cell adhesion, migration, and activation (Singla et al., 2023). These ligand‐dependent pathways suggest that CD47 may connect tissue stress, impaired clearance of injured cells, vascular dysfunction, and chronic cardiac remodeling (Guo et al., 2025). Consistently, CD47 upregulation has been associated with cardiac hypertrophy, fibrosis, myocardial ischemia–reperfusion injury, and atherosclerosis, whereas CD47 inhibition protects cardiac function (Wang et al., 2018). These findings indicate that CD47 is not merely an immune checkpoint molecule but also a stress‐responsive regulator of cardiovascular injury and remodeling.

More importantly, several studies have shown a strong correlation between CD47 expression and cellular aging, with CD47 levels in muscle stem cells increasing with age. As senescence markers such as β‐galactosidase activity rise, the surface CD47 protein density on the cells increases significantly, forming a molecular signature of aging (Porpiglia et al., 2022). Aberrant CD47 expression has also been observed in aged fibroblasts, epithelial cells, and red blood cells (Schloesser et al., 2023; Wang et al., 2020). However, whether CD47 contributes to cardiac aging, particularly doxorubicin‐induced cardiac aging, remains unknown. Given that doxorubicin induces DNA and chromatin damage, oxidative stress, senescence, and cardiac remodeling, CD47 may act as an underexplored link between cellular stress, defective efferocytosis, and age‐like myocardial remodeling.

In this study, we primarily examined the expression profile of CD47 and its role in doxorubicin‐induced heart aging. The ultimate objective was to provide a mitigation strategy for the clinical prevention and treatment of cardiac aging.

## 2 MATERIALS AND METHODS

### 2.1 Reagents and antibodies

Doxorubicin (Catalog Number: HY‐15142) and ligufalimab (Catalog Number: HY‐P99706) were provided by MedChemExpress (China), and the CD47 (Catalog Number: CY5251) and p21 antibodies (Catalog Number: CY5543) were supplied by Abways (China). The SA‐β‐Gal (Catalog Number: ab228562) kit was obtained from Abcam (USA), and the CD47, p16, and p21 primers were provided by Gene Create (China). The PVDF transfer film (Catalog Number: 88518) was provided by Thermo Fisher (USA). The BCA Protein Assay Kit (Catalog Number: P0009) was provided by Beyotime (China). TRIzol (Catalog Number: 9108) was provided by TAKARA (Japan). The cDNA kit (Catalog Number: R412‐01) and SYBR Green mix (Catalog Number: Q713‐02) were provided by Vazyme (China). HRP‐conjugated secondary antibodies (Catalog Number: AB0101/AB0102/AB0103) were supplied by Abways (China).

Ligufalimab, also known as AK117, is a humanized IgG4 monoclonal antibody targeting CD47. It blocks the CD47‐SIRPα interaction and inhibits CD47‐mediated anti‐phagocytic signaling. In this study, ligufalimab was purchased from MedChemExpress and used as an anti‐CD47 blocking antibody in the indicated mouse and cell culture experiments.

### 2.2 Animals

All mice were housed under a 12‐h light/dark cycle in a temperature‐controlled facility at 23 ± 3°C and 30–70% humidity. Six‐month‐old male C57BL/6J mice were obtained from Vital River Laboratory (Beijing, China). The mice were randomly assigned to four groups: control, doxorubicin, ligufalimab, and doxorubicin + ligufalimab. The control group received weekly intraperitoneal injections of 0.9% NaCl. The doxorubicin group received intraperitoneal injections of doxorubicin at 2.5 mg/kg once weekly for six consecutive weeks. The ligufalimab group received intraperitoneal injections of ligufalimab at 1 mg/kg once weekly. The doxorubicin + ligufalimab group received doxorubicin at 2.5 mg/kg together with ligufalimab at 1 mg/kg by intraperitoneal injection once weekly for six consecutive weeks. At the predefined time point after the final dose, echocardiographic assessment was performed, followed by euthanasia and collection of cardiac tissues for subsequent molecular and histological analyses. The standard diet was supplied by Henan Skbes Biotechnology Co., Ltd. (Cat. No. XTI01WC‐010). All animal procedures were conducted in accordance with protocols approved by the Institutional Animal Care and Use Committee (approval no. XYLL‐20220187).

Euthanasia was performed by trained personnel using cervical dislocation in accordance with institutional animal care and ethical guidelines. Mice were gently restrained to minimize distress, and the procedure was carried out rapidly to ensure immediate loss of consciousness. All procedures were supervised by the Animal Ethics Committee and complied with relevant national and institutional regulations. Animal remains were disposed of according to institutional biosafety requirements.

### 2.3 Echocardiographic assessment

After the completion of the drug intervention, echocardiography was performed to evaluate cardiac function. Mice were anesthetized by intraperitoneal injection of 2% pentobarbital sodium solution at 3 mL/kg. The hair on the anterior chest was removed, and the mice were fixed in a supine position with the limbs gently extended. The operation was carried out with a Philips cardiac ultrasound system equipped with an appropriate probe. The cardiac structure and movement of the mice were observed and recorded during the operation, and the measurement kit of the machine was used to measure the left ventricular brachyaxis shortening rate of the mice in each group: Left Ventricular Fractional Shortening (LVFS) and Left Ventricular Ejection Fraction (LVEF).

### 2.4 Cell culture

AC16 cardiomyocytes were cultured in high‐glucose Dulbecco's modified Eagle's medium (DMEM) and divided into four groups: control, Dox, ligufalimab, and Dox + ligufalimab. In the control group, an equal volume of 0.9% NaCl solution was added to the culture dish. The Dox group was treated with 0.1 μM doxorubicin for 48 h. The ligufalimab group was treated with ligufalimab at a concentration of 0.1 μg/mL for 48 h. The Dox + ligufalimab group was treated with doxorubicin (0.1 μM) and ligufalimab (0.1 μg/mL) for 48 h. After treatment, the cells were used for subsequent analyses. The AC16 cell line, originally derived from a male donor, was obtained from the Cell Bank of the Chinese Academy of Sciences.

### 2.5 Bioinformatics data collation

Aging‐related heart tissue sequencing datasets were obtained from the GEO database. The rat heart dataset GSE173360 included five samples per group, comprising young and aged groups. This dataset contained heart tissue sequencing data from 6‐month‐old adult male rats and 24‐month‐old aged male rats. The mouse heart dataset GSE225576 included four samples per group, also comprising young and aged groups. Bioinformatic analysis revealed that CD47 expression was significantly altered in aged heart tissues.

### 2.6 Western blotting

Heart samples were collected and stored at −80°C for later use. The heart tissue was cut into fine fragments, and the tissue and lysate were added to RIPA lysis buffer at a ratio of 100 mg to 1 mL. The mixture was processed in a high‐throughput tissue grinder (20 Hz, 2 min, 3–4 times) and then incubated at 4°C for 30 min to ensure complete lysis. After high‐speed centrifugation, the supernatant, which contained the protein extract, was collected. Protein concentration was determined using the BCA method, and concentrations were normalized across groups. Proteins were transferred to a PVDF membrane using constant‐voltage electrophoresis and a constant‐flow wet transfer method on a 10% SDS‐0 gel. After membrane transfer, the PVDF membrane was incubated in 5% skim milk powder at room temperature. The membrane was then incubated overnight at 4°C with anti‐CD47 (1:1000), anti‐p21 (1:1000), and anti‐β‐actin (1:1000) antibodies. The next day, the membrane was washed three times with PBST solution for 10 min each. Subsequently, the PVDF membrane was incubated with HRP‐labeled sheep anti‐rabbit IgG and HRP‐labeled sheep anti‐mouse IgG solutions for 90 min at room temperature. The membrane was then washed three times with PBST solution, and the target bands were visualized using a chemiluminescence imaging instrument. High‐quality films were selected, and the band intensity of protein expression in each band was determined using ImageJ software. The band intensity of β‐actin expression was used as the baseline for normalization, and the expression levels of the target proteins were analyzed.

### 2.7 Immunofluorescence

For heart tissue staining, paraffin sections were prepared from fixed heart tissues. For cell immunofluorescence staining, AC16 cells were grown on cell slides and fixed with freshly prepared 4% paraformaldehyde for 15 min. Tissue sections or cell slides were washed with PBST for 5 min, permeabilized with PBS containing 0.1% Triton X‐100 for 20 min, and blocked with 2.5% normal goat serum for 1 h. The primary antibody was diluted 1:200 in PBST containing 2.5% normal goat serum and incubated with the slides overnight at 4°C. After washing, the slides were incubated with Alexa Fluor 488‐conjugated goat anti‐rabbit IgG H&L secondary antibody (1:200) for 1 h at room temperature. Non‐immune rabbit IgG was used as a negative control. For tissue samples, three sections were analyzed per mouse, and three random fields were selected from each section for immunofluorescence quantification. For cell samples, three random fields were selected from each slide. Fluorescence images were acquired using a fluorescence microscope, and mean fluorescence intensity was quantified using ImageJ.

### 2.8 RT‐qPCR assay

RNA samples were extracted using TRIzol reagent according to the standard protocol. For each sample, 800 ng of total RNA was reverse transcribed into cDNA using Trans Script All‐in‐One First‐Strand cDNA Synthesis SuperMix (Vazyme Biotech, Nanjing, China). The cDNA was then amplified using the SYBR Green method with the LightCycler® 96 Instrument (Kermey Biotech, Zhengzhou, China). Relative mRNA expression was determined using the 2^−ΔΔCt^ method, with β‐actin as the internal control. The primers used in this study are listed in Table 1.

**TABLE 1:** RT‐qPCR primers.

Name	Species	Sequence	
CD47	Human	Forward	AGAAGGTGAAACGATCATCGAGC
		Reverse	CTCATCCATACCACCGGATCT
CD47	Mouse	Forward	TGGTGGGAAACTACACTTGCG
		Reverse	CGTGCGGTTTTTCAGCTCTAT
P21	Human	Forward	TGTCCGTCAGAACCCATGC
		Reverse	AAAGTCGAAGTTCCATCGCTC
P21	Mouse	Forward	CCTGGTGATGTCCGACCTG
		Reverse	CCATGAGCGCATCGCAATC
P16	Human	Forward	GATCCAGGTGGGTAGAAGGTC
		Reverse	CCCCTGCAAACTTCGTCCT
P16	Mouse	Forward	CGCAGGTTCTTGGTCACTGT
		Reverse	TGTTCACGAAAGCCAGAGCG
β‐Actin	Human	Forward	CATGTACGTTGCTATCCAGGC
		Reverse	CTCCTTAATGTCACGCACGAT
β‐Actin	Mouse	Forward	GTGACGTTGACATCCGTAAAGA
		Reverse	GCCGGACTCATCGTACTCC
KI67	Mouse	Forward	ATCATTGACCGCTCCTTTAGGT
		Reverse	GCTCGCCTTGATGGTTCCT
P53	Mouse	Forward	CCCCTGTCATCTTTTGTCCCT
		Reverse	AGCTGGCAGAATAGCTTATTGAG

### 2.9 Statistical analysis

Data processing and graphing were performed using GraphPad Prism 9. Data are presented as mean ± standard deviation. Comparisons among multiple independent groups were performed using one‐way analysis of variance (ANOVA), followed by appropriate post hoc tests with correction for multiple comparisons. A value of *p* < 0.05 was considered statistically significant.

## 3 RESULTS

### 3.1 CD47 expression in the aged rat and mouse heart

**FIGURE 1:**
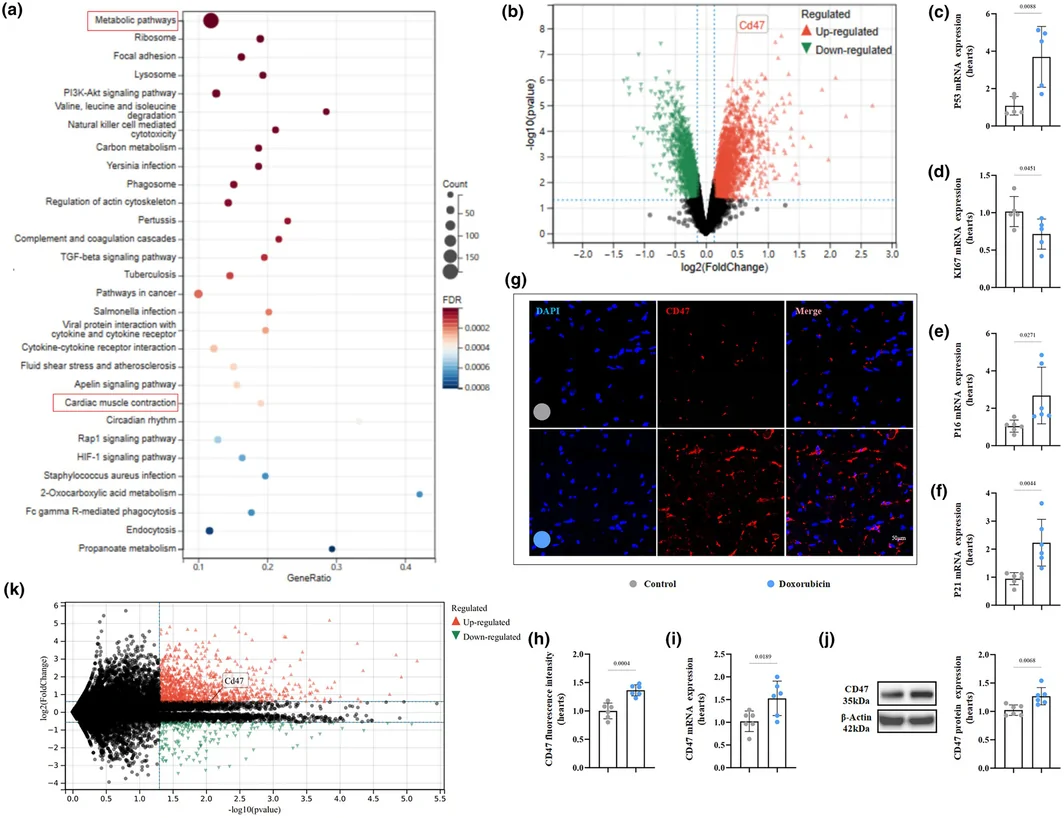
CD47 expression in the aged rat hearts from the GEO database and quantification by q‐PCR and western‐blotting assays in aged mouse hearts. CD47 and Age‐related markers expression in Dox‐induced aged mouse hearts. (a) Enrichment analysis of different gene pathways in the aged rat hearts. (b) Volcano map of differently expressed genes in aged rat hearts. (c) qPCR data showing P53 mRNA expression in aged mouse hearts. (d) qPCR data showing KI67 mRNA expression in aged mouse hearts. (e) qPCR data showing P16 mRNA expression in aged mouse hearts. (f) qPCR data showing P21 mRNA expression in aged mouse hearts. (g) Representative images of CD47 fluorescent staining in control and aged mouse hearts. (h) Quantification of CD47 fluorescent density in aged mouse hearts. (i) qPCR data showing CD47 mRNA expression in aged mouse hearts. (j) Western‐blotting data showing CD47 protein expression in aged mouse hearts. (k) Volcano map of differently expressed genes in aged mouse hearts. *n* ≥ 3. Data are shown as mean ± SD. *p* values are shown in the figure.

Aging‐related cardiac transcriptomic datasets were obtained from the GEO database, including the rat heart dataset GSE173360 and the mouse heart dataset GSE225576. Analysis of GSE173360 showed that CD47 expression was significantly increased in aged rat hearts compared with young controls. Consistently, analysis of the mouse heart dataset GSE225576 demonstrated a similar elevation of CD47 expression in aged mouse hearts. Functional enrichment analysis of differentially expressed genes revealed significant alterations in metabolic and myocardial contraction‐related pathways (Figure 1a,b,k).

### 3.2 Identification of aging models and expression levels of CD47

P53, KI67, p16 and p21 are common aging markers. P21, also known as cyclin‐dependent kinase inhibitor 1A (CDKN1A), and p16, designated p16INK4a, are pivotal tumor suppressor proteins that play essential roles in maintaining cellular homeostasis. The presence of these aging markers was confirmed in both the control and aging heart groups. As illustrated in Figure 1c–f, mRNA levels of P53, KI67, P16 and p21 were markedly elevated in hearts from the Dox group, substantiating the successful construction of the aging model. We next assessed CD47 expression in aged heart tissues. As illustrated in Figure 1g–j, the mRNA and protein expression levels of CD47 in aging heart tissue were significantly increased.

### 3.3 Blockade of CD47 alleviates doxorubicin‐induced cardiac senescence‐like changes and improves cardiac function

**FIGURE 2:**
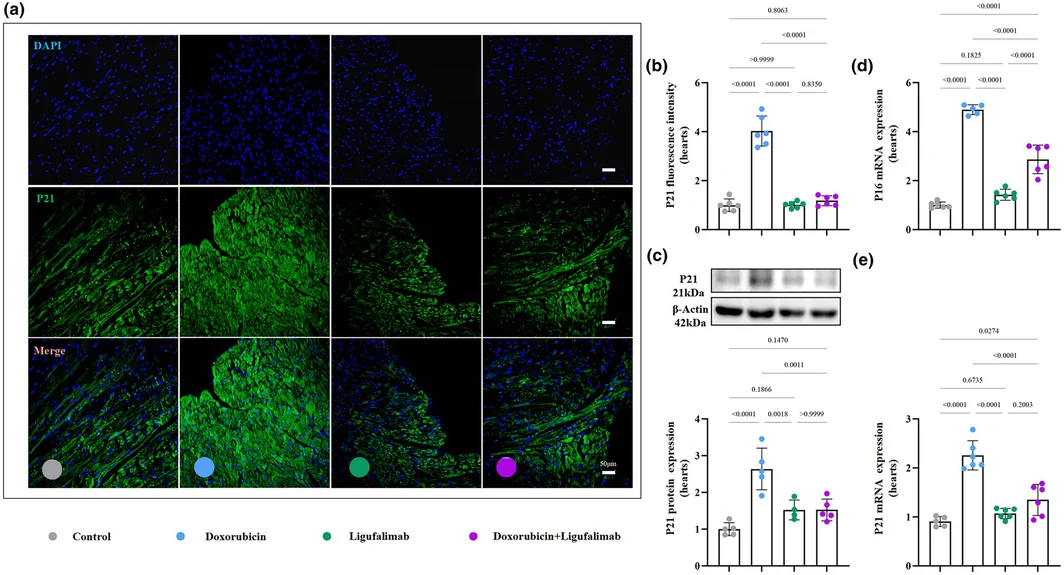
Effect of CD47 inhibition on Dox‐induced mouse heart aging. (a and b) Representative images (a) and quantification (b) of P21 fluorescent staining in the hearts of four groups of mice. (c) Western‐blotting data showing P21 protein expression in the hearts of three groups of mice. (d and E) qPCR data showing P16 (d) and P21 (e) mRNA expression in the hearts of four groups of mice. *n* ≥ 3. Data are shown as mean ± SD. *p* values are shown in the figure.

**FIGURE 3:**
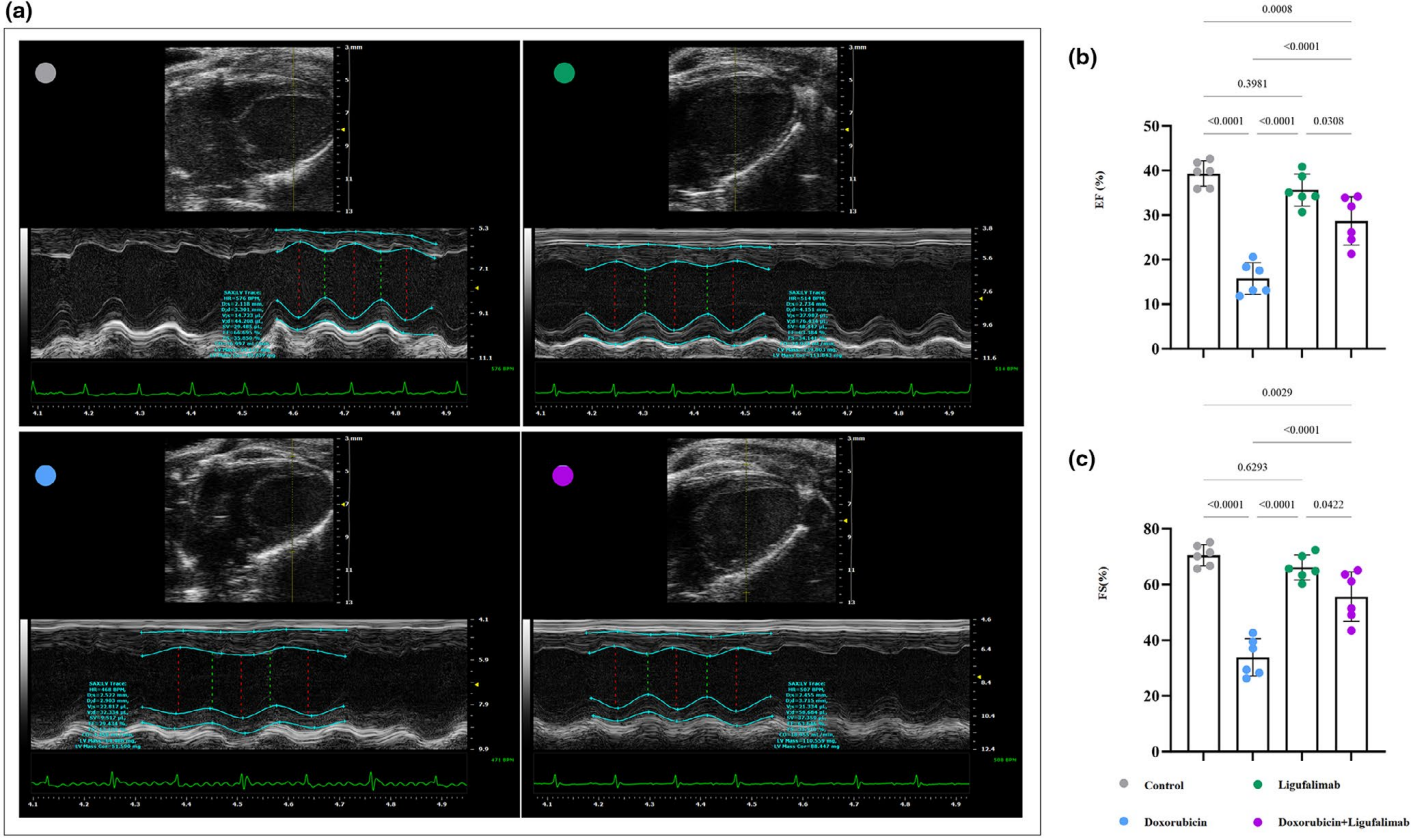
Effect of CD47 inhibition on Dox‐induced cardiac dysfunction. (a) Representative echocardiographic images from control, doxorubicin, ligufalimab, and doxorubicin + ligufalimab groups. (b, c) Quantification of left ventricular ejection fraction (EF) and fractional shortening (FS). *n* ≥ 3. Data are shown as mean ± SD. *p* values are shown in the figure.

Ligufalimab is a CD47‐specific targeting drug that inhibits the function of CD47 without affecting its expression. Administration of ligufalimab to mice to inhibit CD47 resulted in a significant decline in both p21 and p16 expression in the hearts (Figure 2a–e). Echocardiographic data showed that LVFS and LVEF values, indicators of cardiac function, were markedly decreased in the Dox‐treated mice as compared with control mice (Figure 3). However, application of ligufalimab could significantly improve the cardiac function of the Dox‐treated mice (Figure 3).

### 3.4 Blockade of CD47 alleviates doxorubicin‐induced senescence‐like changes in AC16 cardiomyocytes

**FIGURE 4:**
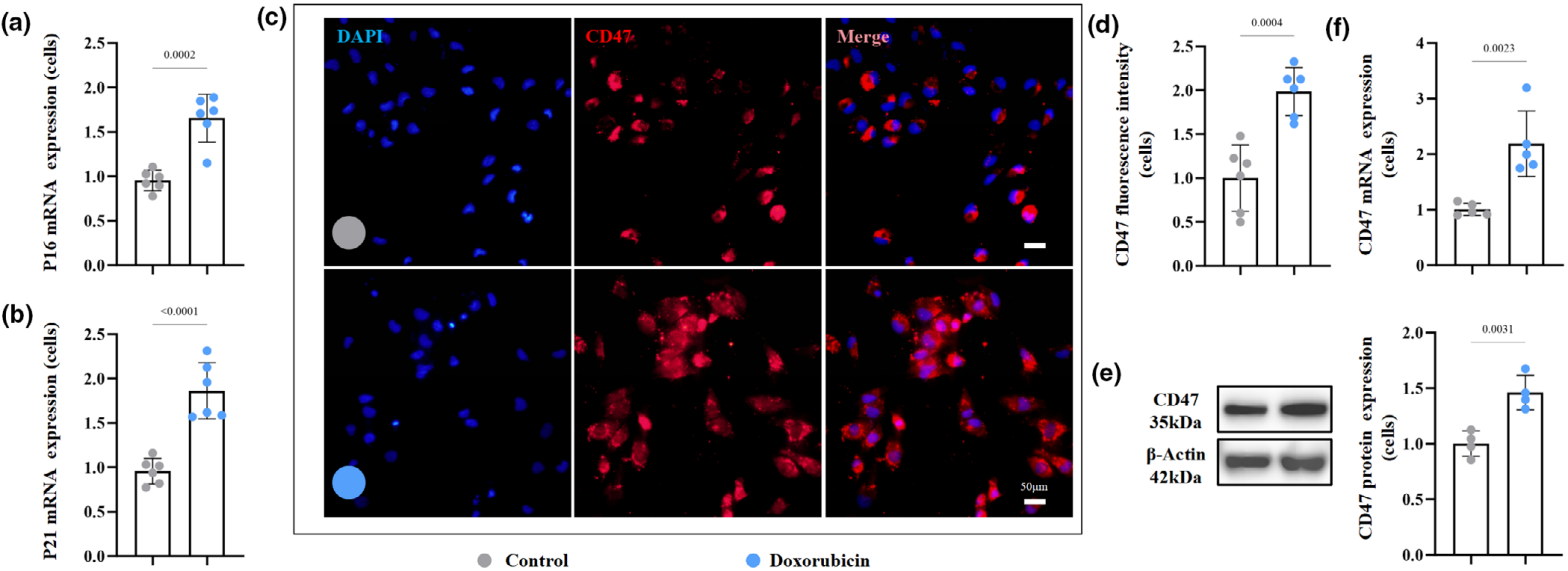
CD47 expression in Dox‐induced senescent cardiomyocytes. (a and b) qPCR data showing P16 (A) and P21 (B) mRNA expression in control and senescent cardiomyocytes. (c) Representative images of CD47 fluorescent staining in control and senescent cardiomyocytes. (d) Quantification of CD47 fluorescent density in senescent cardiomyocytes. (e) Western‐blotting data showing CD47 protein expression in senescent cardiomyocytes. (f) qPCR data showing CD47 mRNA expression in senescent cardiomyocytes. *n* ≥ 3. Data are shown as mean ± SD. *p* values are shown in the figure.

**FIGURE 5:**
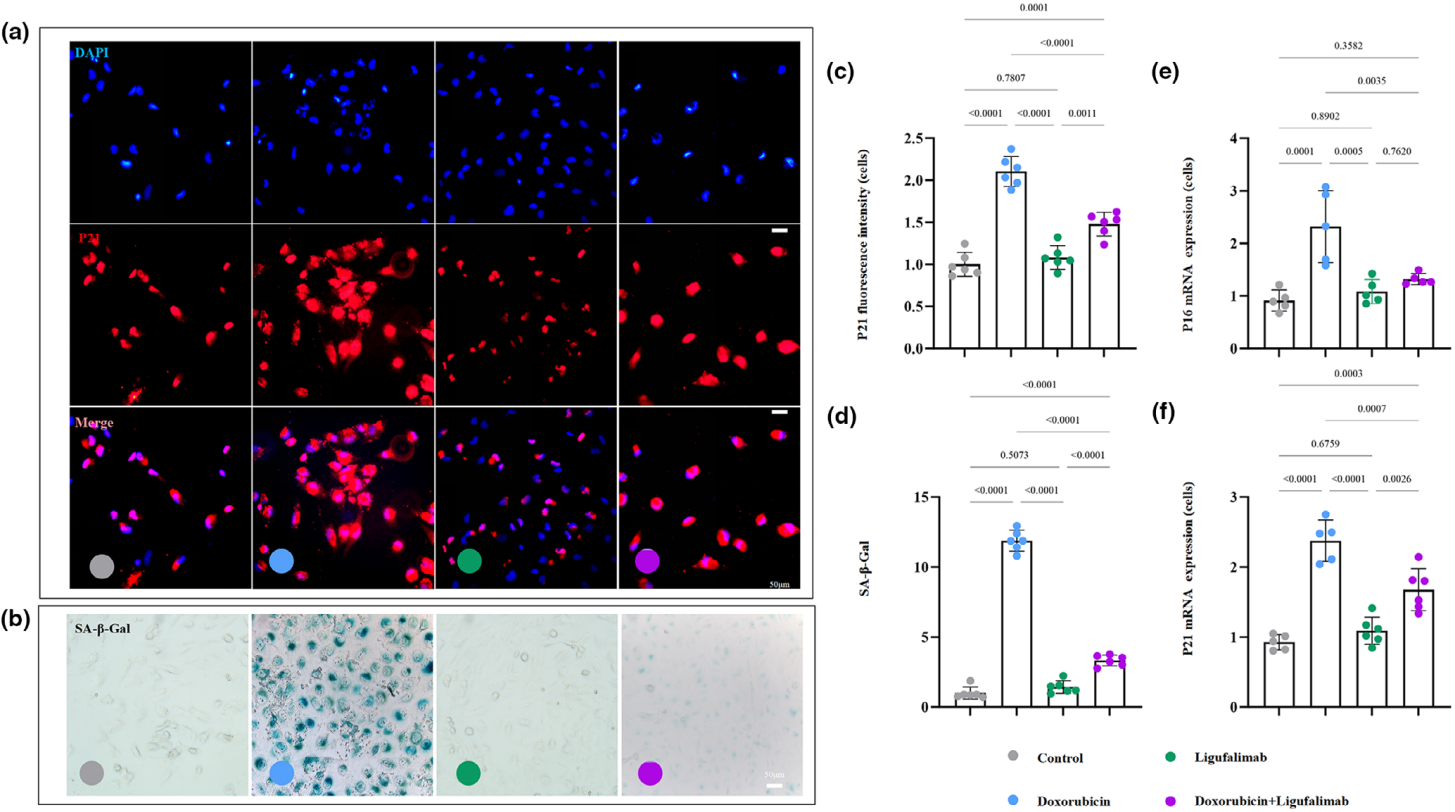
Effect of CD47 inhibition on Dox‐induced cardiomyocyte aging. (a and c) Representative images (a) and quantification (c) of P21 fluorescent staining in the control, Dox‐induced, ligufalimab‐treated, and Dox‐induced + ligufalimab‐treated cardiomyocytes. (b and d) Representative images (b) and quantification (d) of β‐galactosidase staining in four groups of cells. (e and f) qPCR data showing P16 (e) and P21 (f) mRNA expression in four groups of cells. *n* ≥ 3. Data are shown as mean ± SD. *p* values are shown in the figure.

As shown in Figure 4a,b, mRNA levels of p16 and p21 were markedly elevated in cardiomyocytes from the Dox group, substantiating the induction of a senescence‐like phenotype by doxorubicin. Moreover, the mRNA and protein expression levels of CD47 in senescent cardiomyocytes were significantly increased (Figure 4c–f). Following in vitro treatment with ligufalimab to inhibit CD47, the levels of the aging marker p21 were markedly reduced at both the mRNA and protein levels, while p16 levels exhibited a significant decline at the mRNA level (Figure 5a,c,e,f). Concomitantly, a notable reduction in cellular aging was observed (Figure 5b,d).

## 4 DISCUSSION

Cardiac aging is closely associated with the occurrence and progression of myocardial hypertrophy, myocardial remodeling, heart failure, and calcification. This study identified a significant increase in CD47 expression following cardiac aging and demonstrated that blocking CD47 can delay cardiac aging while protecting cardiac function. These findings suggest that CD47 plays a critical role in heart aging and that targeting CD47 may represent a potential therapeutic approach for addressing heart aging.

In recent years, comprehensive research has shown that cardiac aging significantly contributes to the development of cardiomyopathy (Hsieh et al., 2022; Mazevet et al., 2023). Interventions designed to slow the aging process have been shown to reduce its adverse effects on heart function (Kumari et al., 2020; Li et al., 2021; North & Sinclair, 2012). This finding is important because doxorubicin cardiotoxicity involves DNA damage, chromatin disruption, mitochondrial dysfunction, oxidative stress, inflammation, and cellular senescence (Lyu et al., 2007). Our results suggest that CD47 may link these upstream injuries to persistent age‐like cardiac remodeling. The reduction of p53, p16, and p21 after CD47 blockade indicates that CD47 inhibition may attenuate senescence‐associated signaling rather than merely improve cardiac performance. A plausible mechanism involves the CD47–SIRPα axis (Logtenberg et al., 2020). CD47 acts as a “don't eat me” signal by binding SIRPα on macrophages and suppressing phagocytosis and efferocytosis (Kojima et al., 2016). During doxorubicin‐induced cardiac injury, CD47 upregulation may protect stressed or senescent cells from clearance, allowing inflammatory and profibrotic signals to persist in the myocardium. Blocking CD47 may restore the removal of injured or senescent cells, limit chronic inflammatory amplification, and thereby alleviate senescent cardiac remodeling. This interpretation is consistent with the observed reduction in senescence markers and improvement in cardiac function after anti‐CD47 treatment (Guo et al., 2025).

Previous studies have implicated CD47 in vascular injury, atherosclerosis, myocardial ischemia–reperfusion injury, tissue ischemia and age‐related metabolic dysfunction (Hernández‐Mercado et al., 2021; Kojima et al., 2016; Olsson & Oldenborg, 2008). In the present study, bioinformatic analyses from both rat (GSE173360) and mouse (GSE225576) cardiac aging datasets consistently revealed increased CD47 expression in aged hearts, suggesting that CD47 upregulation represents a conserved feature associated with cardiac aging across species. Our study extends these observations by identifying CD47 as a regulator of doxorubicin‐induced cardiac aging. The ability of CD47 blockade to preserve cardiac function in doxorubicin‐treated mice and suppress senescence responses in human cardiomyocytes suggests that CD47 may integrate cardiovascular aging, immune clearance and cellular stress signaling.

Ligufalimab is an anti‐CD47 antibody. Our data suggest that ligufalimab, or other anti‐CD47 strategies, may have therapeutic potential for alleviating doxorubicin‐induced cardiac aging. Its efficacy in both mouse models and cultured human cells supports the translational relevance of CD47 blockade and reduces the likelihood that the protective effect is species‐specific or model‐dependent. However, clinical translation will require careful optimization of treatment timing, dose, tissue specificity, and safety, given the broad expression of CD47 and its role in immune surveillance and cell clearance. Future studies should determine whether transient or cardiac‐targeted CD47 inhibition can retain cardioprotective effects while minimizing systemic adverse effects (Habicht et al., 2025; Qu et al., 2022).

Several limitations should be acknowledged. First, although our data support a protective role of CD47 blockade, the downstream mechanisms remain incompletely defined. Further studies should directly assess SIRPα‐dependent efferocytosis, thrombospondin‐1/CD47 signaling, mitochondrial function, ROS production, inflammatory cytokines, and senescence‐associated secretory factors. Second, the relative contributions of cardiomyocytes, endothelial cells, fibroblasts, and cardiac immune cells to CD47‐mediated aging require clarification. Cell‐specific genetic models, macrophage‐depletion experiments, and efferocytosis assays would help determine whether the benefits of anti‐CD47 therapy are immune‐mediated, cardiac‐cell‐intrinsic, or both. Third, although cultured human cells increase the translational relevance of our findings, additional human cardiac models and long‐term in vivo studies are needed to evaluate durability, safety, and therapeutic windows.

In conclusion, this study suggests that CD47 is a potential marker of aging and an important contributor to doxorubicin‐induced cardiac aging. Blocking CD47 can improve doxorubicin‐induced cardiac dysfunction and delay heart aging. A significant limitation of this study is that it only focuses on cardiac aging, and further investigations will be conducted to determine whether CD47 also plays a role in the aging process of other organs.

## AUTHOR CONTRIBUTIONS

**Aiwei Yan:** Data curation; formal analysis; investigation; methodology. **Rui Mai:** Formal analysis; investigation; methodology. **Ting Cao:** Data curation; methodology. **Changen Duan:** Resources; validation. **Sheng Liu:** Methodology; resources; visualization. **Xianwei Wang:** Conceptualization; funding acquisition; supervision.

## FUNDING INFORMATION

This work was supported by Central Plains Science and Technology Innovation Leading Talents (244200510003), and the Henan Provincial Joint Fund for Scientific and Technological Research (232301420102).

## CONFLICT OF INTEREST STATEMENT

The authors have nothing to report.

## ETHICS STATEMENT

XYLL‐20220568 (Xinxiang Medical University Ethics Committee).

## Data Availability

The data used in this study can be provided by the corresponding author upon reasonable request. If public data were used in the research, the relevant data were all sourced from public databases or publicly available materials. The specific sources have been indicated in the main text or the references.
